# An Eye-Tracking Study on Text Accessibility and Comprehension in University Students

**DOI:** 10.3390/bs16061041

**Published:** 2026-06-22

**Authors:** Sergio Navas-León, Jon Andoni Duñabeitia

**Affiliations:** Centro de Investigación Nebrija en Cognición (CINC), Department of Education, Universidad Nebrija, 28043 Madrid, Spain; snavas@nebrija.es

**Keywords:** accessibility, adults, cognition, education, reading, university

## Abstract

Easy-to-Read (E2R) recommendations aim to improve accessibility, but it remains unclear whether some visual and typographic adaptations may also benefit readers without disabilities. This study examined the effects of different text formats on reading comprehension and visual processing in university students using eye-tracking. Twenty-four young adults without cognitive disabilities read texts presented in three formats: hard-to-read, control, and Easy-to-Read. Reading comprehension was assessed with multiple-choice questions, and eye movements were recorded during reading. Data were analyzed using linear mixed-effects models. Text Format significantly affected reading comprehension, with estimated accuracy highest in the E2R format and significantly higher than in the hard-to-read format. The E2R format was also associated with shorter fixation durations and larger saccades than the other formats, suggesting a pattern compatible with a reduced cognitive demand in some eye-movement measures. Fixation count was highest for hard-to-read texts and significantly higher than in the control format, whereas differences involving E2R were not significant. Reading time showed a trend towards significance, with descriptively longer reading times for hard-to-read texts than for the control and E2R formats. These findings suggest that E2R adaptations, originally developed to support populations with cognitive needs, may also facilitate comprehension and reading efficiency in readers without cognitive disabilities.

## 1. Introduction

Digital reading has become central to everyday life and to access to information, education, health, and public services (Leu et al., 2015). As a result, the accessibility of online content is increasingly considered both a usability issue and a matter of equity and rights, as reflected in the European Accessibility Act (European Parliament and Council of the European Union, 2019) and Article 21 of the UN Convention on the Rights of Persons with Disabilities (United Nations, 2006). Accessibility is especially relevant for people with disabilities (e.g., individuals with dyslexia, intellectual disabilities, or age-related cognitive decline), older adults, and other groups with cognitive or sensory vulnerabilities (Droutsas et al., 2025; Henni et al., 2022; Mason et al., 2021; Van Der Geest & Velleman, 2014). In this context, E2R has emerged as an important framework for promoting accessible communication (Fărcașiu et al., 2023).

E2R recommendations typically combine linguistic and visual principles, such as the use of frequent words, simpler syntactic structures, pictograms or icons, and accessible layout features including sans-serif fonts and left-aligned text (González-Sordé & Matamala, 2024). These recommendations overlap with broader accessibility standards for digital content, particularly the Web Content Accessibility Guidelines (WCAG), which have also been incorporated into European and Spanish standards (Asociación Española de Normalización [UNE], 2018; World Wide Web Consortium [W3C], 2022).

From a psycholinguistic perspective, some E2R recommendations are relevant because they may affect basic perceptual and attentional processes involved in reading. Research has shown, for example, that sans-serif fonts are generally more legible on screens than serif fonts, especially for people with visual difficulties (Bernard et al., 2001; British Dyslexia Association, 2012; Moret-Tatay & Perea, 2011; Russell-Minda et al., 2007). Other typographic variables, such as line spacing, pictograms, text alignment, inter-letter spacing, and contrast, have also been associated with changes in readability and visual processing (Conlon & Sanders, 2011; Ling & van Schaik, 2007; Perea & Gomez, 2012a, 2012b; Perea et al., 2016; Rello & Marcos, 2012; Rello et al., 2013; Rivero-Contreras et al., 2021, 2023). These mechanisms are not limited to clinical populations, as they reflect general visual and cognitive processes underlying reading.

However, although institutional interest in E2R has grown considerably, empirical evidence regarding its effectiveness remains limited and heterogeneous (e.g., Fărcașiu et al., 2023; González-Sordé, 2025; González-Sordé et al., 2026; Hurtado et al., 2014; Iacono, 2026; Rivero-Contreras & Saldaña, 2020; Sutherland & Isherwood, 2016). In addition, research on fluency manipulations in university students suggests that easier presentation does not always lead to better learning outcomes (Diemand-Yauman et al., 2011), and some typographic manipulations have shown null effects in skilled adult readers (Duñabeitia et al., 2023).

In this context, it could be argued that existing evidence suggests that E2R adaptations may affect reading processing, although their effects on reading comprehension remain mixed. Furthermore, there is a call in the field for applying these adaptations more broadly, as improving content accessibility may benefit all users, not only those with specific impairments (Henni et al., 2022; Kurt, 2019; Schmutz et al., 2016, 2017, 2019). However, empirical evidence supporting these benefits in the general population is still limited (Ekin et al., 2025) and especially for visual and typographic features, as highlighted in a recent systematic review on the topic (Iacono, 2026).

Accordingly, the aim of the present study was to examine the effects of different text formats on reading comprehension and eye-movement measures in young Spanish university students without cognitive disabilities. To do so, we compared three text presentation formats, hard-to-read, control, and E2R, by manipulating visual and typographic variables commonly highlighted in E2R and accessibility guidelines (e.g., font type, text alignment, interline spacing, inter-letter spacing, paragraph spacing, and contrast) (Inclusion Europe, 2020; World Wide Web Consortium [W3C], 2022). These features were combined to reflect how the E2R format is typically implemented in real-world accessible materials. We assessed reading comprehension, fixation count, average fixation duration, average saccade amplitude, and reading time. Eye tracking was used because it provides sensitive measures of reading behavior and cognitive effort during text processing (Scaltritti et al., 2019).

Based on previous research on text accessibility and visual processing, we formulated two confirmatory hypotheses. First, we expected that participants would obtain higher reading comprehension and shorter reading times in the E2R format than in the hard-to-read format, or at least performance comparable to the control format. Second, we expected the E2R format to be associated with shorter fixation durations, fewer fixations, and larger saccade amplitudes than the hard-to-read format, or at least with eye-movement patterns comparable to those observed in the control format, reflecting a pattern compatible with reduced processing demands during reading (Krejtz et al., 2018; Palinko et al., 2010; Wang et al., 2024; Zagermann et al., 2016).

## 2. Materials and Methods

### 2.1. Participants

Participants were students from Universidad Nebrija in Madrid, Spain, who volunteered to participate and were unaware of the study purpose. Twenty-four students (M = 23.74, SD = 4.15; range = 18–31 years) participated in the experiment, of whom 19 were female. Recruitment took place between April and July 2025 through the laboratory’s internal platform, drawing participants from a pool of individuals registered to take part in experiments. Inclusion criteria were: (a) normal or corrected-to-normal visual acuity, (b) being a young adult aged between 18 and 35 years, and (c) obtaining an age- and sex-adjusted score within the normal range on the Cognitive Assessment Battery (CAB™) PRO (CogniFit Inc., San Francisco, CA, USA), which provides a standardized score from 0 to 100. In the present sample, the mean CAB™ PRO score was 64.45 (SD = 14.18), and no participant was excluded based on this screening. Exclusion criteria included: (a) self-reported history of visual and ocular pathology, neurological disorders, or significant brain injury; (b) reported history of mental illness; (c) current use of recreational drugs; (d) incomplete data collection or unsuccessful eye-tracking system calibration; and (e) non-native Spanish speakers.

Simulation-based model-aligned power analyses were conducted in R version 4.5.3 (R Core Team, 2024) to evaluate whether the sample size provided sufficient power for the mixed-effects modeling approach used for the five primary outcomes: reading comprehension, reading time, fixation count, average fixation duration, and average saccade amplitude. For the binomial comprehension model, power was estimated using the *simr* package (Green & MacLeod, 2016). For the linear mixed-effects models, robust Monte Carlo simulations were implemented using the fitted *lme4* model objects (Bates et al., 2015). For each outcome-specific final model, power was estimated for the global effect of Text Format by comparing the fitted model with a reduced model that preserved the same random-effect structure but removed Text Format. This procedure incorporated the observed three-condition within-subject design, the number of participants, the number of trials, the fixed-effect estimates used as simulation-generating values, and the random effects and residual variance structure of each model.

For reading comprehension, the dependent variable was binary at the item level, coded as 0 = incorrect and 1 = correct; therefore, a binomial mixed-effects model with a logit link was used. The final model included Text Format as a fixed effect and random intercepts for Participant and Trial. The simulation-based analysis indicated 79.6% power for the global Text Format effect, 95% CI [77.0, 82.1], with 24 participants. For the linear mixed-effects models, estimated power was 60.5% for reading time, 95% CI [57.4, 63.5], 90.2% for fixation count, 95% CI [88.2, 92.0], 100% for average fixation duration, 95% CI [99.6, 100], and 100% for average saccade amplitude, 95% CI [99.6, 100]. Thus, the sample size provided adequate power for reading comprehension and the main eye-tracking outcomes, although power for reading time was more limited.

The study adhered to the standards of the Declaration of Helsinki, and the procedure was approved by the ethics committee of Universidad Nebrija (approval code UNNE-2022-0017). All participants were informed about the experiment, given time to ask questions, and asked to sign informed consent forms.

### 2.2. Materials

Three fictional countries were created. Each country included 15 texts covering topics such as population and demographic trends, history, economy, politics, energy, education system, tourism, language, religion, cuisine, natural landscapes, healthcare systems, infrastructure, climate, and geography, resulting in a total of 45 texts. The texts were developed through an iterative process to reduce textual complexity and ensure accessibility. Multiple versions of each text were refined, focusing on sentence structure, readability, and clarity. During this process, complex sentences were simplified, jargon was reduced, and content was adapted for a broad audience. Simplification strategies included shortening lengthy sentences and minimizing the use of adverbs and superlatives (for the full set of texts, see OSF project: https://osf.io/25ar8/overview?view_only=6cc4e8abaaab487fada9de1e7e031b94, accessed on 1 May 2026).

Furthermore, to ensure comparability across texts, word frequency was controlled using EsPal (Duchon et al., 2013). Across the 871 analyzed words, no significant differences were found between countries in Zipf frequency values, F(2, 611) = 2.11, *p* = 0.122. At the text level, the number of words also did not differ significantly across countries, F(2, 42) = 2.84, *p* = 0.070. A further analysis using INFLESZ (Barrio-Cantalejo et al., 2008) showed that the mean number of syllables per word ranged from 2.07 to 2.18, and the mean number of words per sentence ranged from 11.13 to 11.54. No significant between-country differences were observed in lexical density (proportion of lexical content words relative to total words), F(2, 42) = 0.85, *p* = 0.433, lexical diversity (number of unique words divided by the total number of words), F(2, 42) = 0.10, *p* = 0.908, or words per sentence, F(2, 42) = 0.11, *p* = 0.899.

The study used a within-subject design with three text formats: hard-to-read, control, and E2R, such that each participant read texts in all three formats. Six counterbalanced lists were used to rotate the assignment of text formats across countries, ensuring that each text appeared equally often in each format across participants.

**Figure 1:**
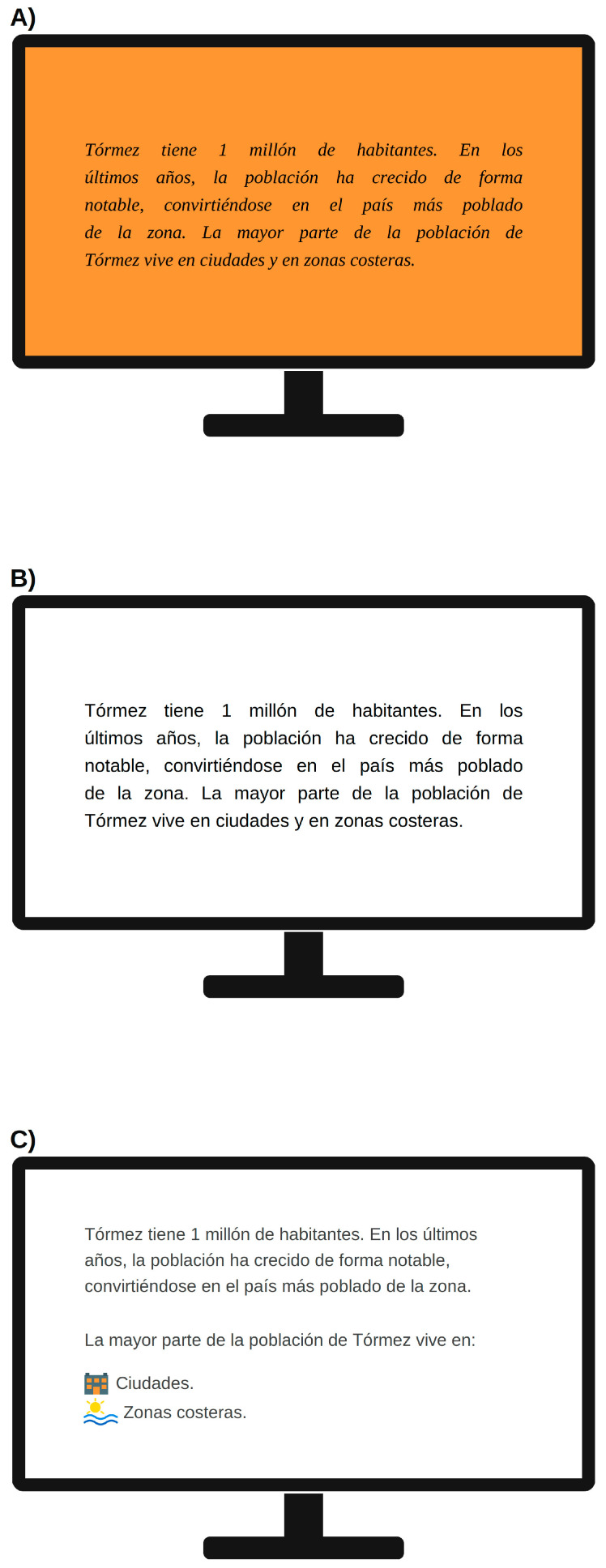
Examples of the three text formats: panel (**A**) corresponds to the hard-to-read format, including an orange background and a serif font in italics; panel (**B**) corresponds to the control format, with sans serif black font on a white background; and panel (**C**) corresponds to the E2R format. Translation: Tórmez has one million inhabitants. In recent years, the population has grown notably, making it the most populated country in the area. Most of the population of Tórmez lives in cities and coastal areas.

In the hard-to-read format (see Figure 1A), a serif font (Lucida Calligraphy) was used (font size = 18), with fully justified text, no paragraph breaks, 1.5-line spacing, default word spacing, and a high contrast ratio (background: EA953B; font color: #000000). In the control format (see Figure 1B), a sans-serif font (Lucida Sans) was used (font size = 18), with fully justified text, no paragraph breaks, 1.5-line spacing, default word spacing, and an adequate contrast ratio (background: #FFFFFF; font color: #000000). In the E2R format (see Figure 1C), a sans-serif font (Lucida Sans) was used (font size = 18), with left-aligned text, 1.5-line spacing, increased paragraph spacing (twice the font size), and word spacing of at least 0.16 times the font size. An optimized contrast ratio was applied (background: FFFFFF; font color: #404040), and color pictograms were included to support information processing.

A multiple-choice questionnaire was designed to assess reading comprehension of the content associated with each country. The three questionnaires comprised 90 items in total (30 per country). The order of country presentation was counterbalanced across participants, whereas the order of questions and response options was randomized for each participant. Each question included four response options, one correct answer, two distractors based on information from other countries to increase task difficulty, and one option designed to assess plausibility and detect inconsistent or unrealistic choices. The full set of questionnaire items is available in the OSF project: https://osf.io/25ar8.

### 2.3. Procedure

Participants were seated in a room with constant lighting and temperature. The experimental session lasted approximately one hour. Before starting, participants were informed that the study concerned learning, that their participation was voluntary, and that they could withdraw at any time. Written informed consent was obtained prior to participation.

At the beginning of the experiment, participants provided basic sociodemographic information, including sex assigned at birth and age, and completed the cognitive assessment.

Later, participants were seated in front of the eye-tracking system, facing a 27″, 165 Hz LED monitor (Lenovo Legion Y27h-30; Lenovo Group Limited, Beijing, China) with a resolution of 1920 × 1080 pixels. Eye movements were recorded using the EyeLink Portable Duo system (SR Research, Kanata, ON, Canada), which tracked both eyes at a sampling rate of 500 Hz using the dark pupil–corneal reflection method. A height-adjustable chinrest was used to maintain head stability and a fixed viewing distance of 98 cm. A nine-point calibration and validation procedure was conducted individually for each participant. Calibration was accepted when the average validation error was below 0.5° of visual angle and the maximum error did not exceed 1.0°. When these criteria were not met, the calibration procedure was repeated. Drift correction was applied throughout the experiment.

Once calibrated, participants began the reading phase. They were instructed to read each text silently and advance to the next text by pressing the spacebar once they had finished reading. Before the experimental trials, participants completed five practice trials, which were used to familiarize them with the procedure and to resolve any questions. Once these practice trials had been completed, participants were presented with texts corresponding to different countries and text formats (hard-to-read, control, E2R). Each trial began with a fixation cross presented at the center of the screen for 500 ms. This was followed by presentation of the text, which remained on screen for a maximum duration of 30,000 ms or until the participant pressed the spacebar to continue. Once a text was completed, it was no longer possible to return to it.

After completing the reading phase, participants were offered a 5 min break. They then performed a Flanker task (Eriksen & Eriksen, 1974), a cognitive control task in which they indicated the direction of a central arrow while ignoring surrounding distractor arrows. This task was included to minimize potential recency effects before reading comprehension questionnaires. The duration of the flanker task was identical for all participants (60,000 ms).

Participants then completed three reading comprehension questionnaires (one per country), presented in counterbalanced order across participants, which together included 90 multiple-choice questions administered on a tablet device (Lenovo Tab M10 HD, model TB-X306F; Lenovo Group Limited, Beijing, China). Each question was based on the texts read during the experiment and required participants to select the correct response from four options. Participants completed this phase at their own pace.

At the end of the experiment, participants were debriefed, provided with a summary information sheet, and thanked for their participation.

### 2.4. Data Analysis

Dependent variables were reading comprehension, fixation count, average fixation duration, average saccade amplitude, and reading time, computed across the full text to capture global reading behavior, rather than being restricted to predefined AOIs. Reading comprehension was analyzed as a binary outcome (correct vs. incorrect). Reading time was defined as the interval from text onset to keypress.

Data was pre-processed prior to modeling. A fixation was operationally defined as a relatively stable period of gaze during which successive samples remained spatially clustered long enough to be classified as a fixation by the I-DT algorithm. In the present study, fixations were identified using the I-DT algorithm with a minimum duration of 100 ms. Fixation count corresponded to the number of valid fixations recorded during each text-reading trial, and average fixation duration corresponded to the mean duration, in milliseconds, of valid fixations during that trial. A saccade was operationally defined as a rapid eye movement between two successive fixation periods. Saccades were detected using velocity and acceleration thresholds of 30°/s and 8000°/s^2^, respectively, and saccades shorter than 2° were excluded (Holmqvist et al., 2011). Average saccade amplitude corresponded to the mean angular distance, in degrees of visual angle, of valid saccades during each text-reading trial. Reading time was defined as the interval from text onset to the Participant’s keypress indicating that they had finished reading the text. Outliers were identified separately for each dependent variable within each Participant × Text Format cell using a ±2.5 SD criterion and re-coded as missing values. Practice trials were excluded from the analysis.

**Figure 2:**
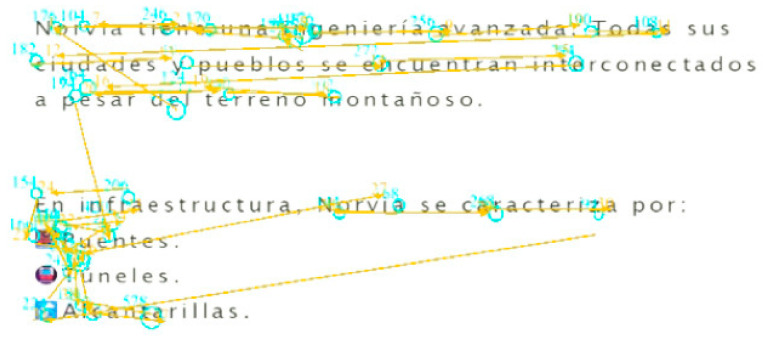
Representative gaze plot overlaid on an experimental reading stimulus. Blue circles indicate fixations, numbers show fixation duration in milliseconds, and arrows indicate the direction of successive saccadic movements. The figure illustrates one participant and one trial; analyses were conducted on trial-level measures across all participants and trials.

To facilitate interpretation of the eye-movement measures, Figure 2 presents a representative gaze plot from one participant during one reading trial. The figure illustrates the sequence of fixations and saccades overlaid on the stimulus text and provides a visual example of the type of eye-movement information from which fixation count, average fixation duration, and average saccade amplitude were derived.

Because comprehension responses were binary and collected at the item level, a binomial mixed-effects model (GLMM) was used instead of aggregated accuracy scores or ordinary linear models. This approach allowed us to model the probability of a correct response while accounting for the non-independence of repeated observations within participants and across trials/items. For the eye-movement outcomes, linear mixed-effects models (LMMs) were fitted for fixation count, average fixation duration, and average saccade amplitude, whereas reading time was analyzed using an LMM fitted to log-transformed reading times. Overall, mixed-effects modeling reflected the fully within-subject repeated-measures structure, in which each of the 24 participants contributed multiple observations across the three text-format conditions (90 observations for comprehension and 45 observations for eye-movement outcomes).

All models initially considered Text Format, Trial Order, and their interaction as the maximal fixed-effect structure, together with simpler nested fixed-effect structures derived from it. Random-effect structures were specified following the general recommendation to account for both participant and trial variability and to consider random slopes when supported by the data (Barr et al., 2013). Accordingly, the candidate model included random intercepts for Participant and Trial, random slopes for Text Format, Trial Order, and, where applicable, their interaction, as well as simpler random-effect structures derived from this maximal specification.

For each outcome, model comparison followed a sequential procedure. Models that did not converge or produced singular fits were first excluded. The remaining models were compared with their corresponding null models using likelihood-ratio tests, retaining only those that improved model fit. Random slopes unsupported by the retained fixed-effect structure or leading to unnecessary model complexity were discarded. Model selection then favored parsimonious and interpretable models, with BIC used as the main criterion to balance model fit and complexity. When supported by model fit, models including Text Format were prioritized, given its relevance to the study hypotheses. Across outcomes, preference was given to random-effect structures including random intercepts for Participant and Trial.

Pairwise comparisons were conducted using estimated marginal means with Bonferroni correction when the retained model included Text Format as a predictor. Model diagnostics were performed using DHARMa residual diagnostics, including dispersion and zero-inflation tests where applicable (Hartig et al., 2024).

Analyses were conducted in R version 4.5.3 (R Core Team, 2024). LMMs were fitted using lme4 (Bates et al., 2015), with *lmerTest* (Kuznetsova et al., 2017) used for inference in linear mixed models. Estimated marginal means and Bonferroni-adjusted pairwise comparisons were obtained with *emmeans* (Lenth et al., 2019), and model diagnostics were conducted using DHARMa (Hartig et al., 2024). Statistical significance was set at *p* < 0.05 for all analyses.

## 3. Results

Simulated residual analyses showed no evidence of dispersion or zero-inflation problems. Dispersion estimates were close to 1 and non-significant across reading comprehension, fixation count, average fixation duration, average saccade amplitude, and log-transformed reading time (ps ≥ 0.830), while zero-inflation tests were also non-significant (ps = 1). Results of the mixed-effects models are reported below.

Across all five outcome variables, the retained random-effect structure consisted of random intercepts for Participant and Trial only; random slopes did not improve model fit, led to non-convergence, or produced singular fits in all cases and were therefore excluded.

### 3.1. Reading Comprehension

The best-fitting model retained Text Format as a fixed effect, with random intercepts for Participant and Trial:

Reading comprehension ~ Text Format + (1|Participant) + (1|Trial)

Raw participant-level accuracy was highest in the E2R format, M = 66.96%, SD = 22.13, followed by the control format, M = 61.53%, SD = 22.20, and the hard-to-read format, M = 60.00%, SD = 23.75. The model showed a significant global effect of Text Format, *p* = 0.011. The likelihood-ratio comparison against the corresponding null model also supported this effect, χ^2^(2) = 9.21, *p* = 0.010. The model explained a small proportion of variance through fixed effects alone, marginal R^2^ = 0.006, while the full model including random effects showed conditional R^2^ = 0.208.

**Figure 3:**
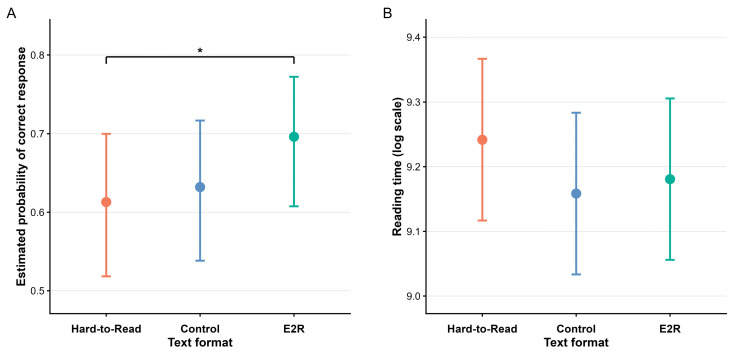
Panel (**A**) shows the estimated probability of correct responses, and panel (**B**) shows reading time (log scale). Error bars represent 95% confidence intervals. The number of asterisks reflects the adjusted *p*-value threshold: * *p* < 0.05.

Estimated marginal probabilities indicated that reading comprehension was highest in the E2R format, 69.6%, 95% CI [60.8, 77.2], followed by the control format, 63.2%, 95% CI [53.8, 71.7], and the hard-to-read format, 61.3%, 95% CI [51.8, 70.0] (see Figure 3A). Pairwise comparisons showed that the E2R format produced significantly higher comprehension accuracy than the hard-to-read format, log-odds estimate = 0.369, SE = 0.128, Bonferroni-adjusted 95% CI [0.062, 0.676], OR = 1.45, 95% CI [1.06, 1.97], *p* = 0.012. The comparison between control and E2R showed a trend towards significance after Bonferroni correction, estimate = −0.288, SE = 0.129, Bonferroni-adjusted 95% CI [−0.597, 0.021], OR = 0.75, 95% CI [0.55, 1.02], *p* = 0.077. The control and hard-to-read formats did not differ significantly, estimate = 0.081, SE = 0.125, Bonferroni-adjusted 95% CI [−0.218, 0.380], OR = 1.08, 95% CI [0.80, 1.46], *p* = 1.00. A graphical representation of these Bonferroni-adjusted pairwise comparisons is provided in the Supplementary Materials (Figure S1).

### 3.2. Reading Time

The best-fitting model retained the fixed effects of Text Format and Trial Order, with random intercepts for Participant and Trial:

log(Reading Time) ~ Text Format + Trial Order + (1|Participant) + (1|Trial)

Raw reading time in milliseconds was descriptively highest in the hard-to-read format, M = 11,133 ms, SD = 3428.81, followed by the E2R format, M = 10,402.98 ms, SD = 2995.72, and the control format, M = 10,261 ms, SD = 3339.38. The model showed a trend towards significance for the global effect of Text Format, *p* = 0.076, and a significant effect of Trial Order, β = −0.036, SE = 0.007, 95% CI [−0.050, −0.023], *p* < 0.001. The model showed marginal R^2^ = 0.018 and conditional R^2^ = 0.678.

Estimated marginal means for log-transformed reading time were highest in the hard-to-read format, M = 9.24, SE = 0.06, 95% CI [9.12, 9.37], followed by the E2R format, M = 9.18, SE = 0.06, 95% CI [9.06, 9.31], and the control format, M = 9.16, SE = 0.06, 95% CI [9.03, 9.28] (see Figure 3B). Pairwise comparisons showed that the control and E2R formats did not differ significantly, estimate = −0.022, SE = 0.038, Bonferroni-adjusted 95% CI [−0.114, 0.070], d = −0.10, 95% CI [−0.53, 0.32], *p* = 1.00. The control and hard-to-read formats showed a trend towards significance after Bonferroni correction, estimate = −0.083, SE = 0.038, Bonferroni-adjusted 95% CI [−0.175, 0.009], d = −0.38, 95% CI [−0.81, 0.04], *p* = 0.090. The E2R and hard-to-read formats did not differ significantly, estimate = −0.061, SE = 0.038, Bonferroni-adjusted 95% CI [−0.153, 0.031], d = −0.28, 95% CI [−0.70, 0.14], *p* = 0.329. A graphical representation of these Bonferroni-adjusted pairwise comparisons is provided in the Supplementary Materials (Figure S2).

### 3.3. Eye-Movement Measures

#### 3.3.1. Fixation Count

The best-fitting model retained the fixed effects of Text Format and Trial Order, with random intercepts for Participant and Trial:

Fixation Count ~ Text Format + Trial Order + (1|Participant) + (1|Trial)

Raw fixation count was highest in the hard-to-read format, M = 44.92, SD = 13.74, followed by the E2R format, M = 41.74, SD = 10.91, and the control format, M = 40.04, SD = 11.22. The model showed a significant global effect of Text Format, *p* = 0.026, and a significant effect of Trial Order, β = −1.521, SE = 0.321, 95% CI [−2.152, −0.891], *p* < 0.001. The model showed marginal R^2^ = 0.022 and conditional R^2^ = 0.589.

**Figure 4:**
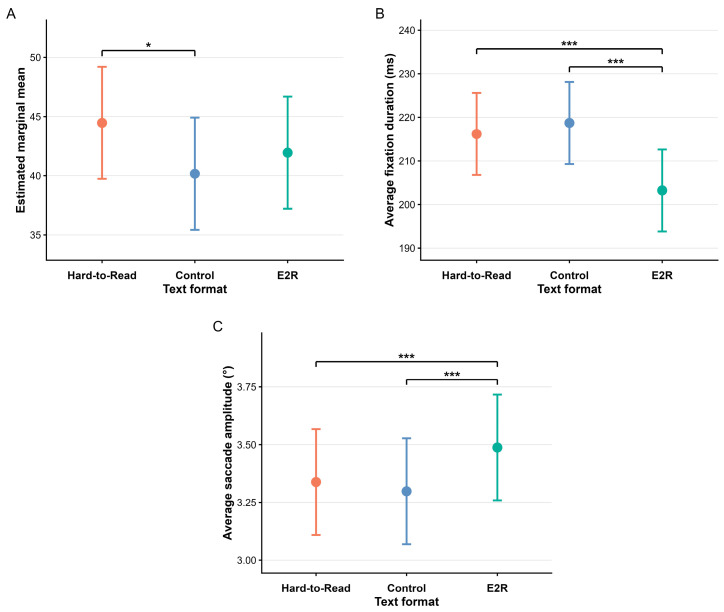
Eye-movement effects of Text Format. Panel (**A**) shows fixation count, panel (**B**) shows average fixation duration, and panel (**C**) shows average saccade amplitude. Error bars represent 95% confidence intervals. The number of asterisks reflects the adjusted *p*-value threshold: * *p* < 0.05, *** *p* < 0.001.

Estimated marginal means indicated that fixation count was highest in the hard-to-read format, M = 44.47, SE = 2.34, 95% CI [39.74, 49.20], intermediate in the E2R format, M = 41.95, SE = 2.34, 95% CI [37.22, 46.69], and lowest in the control format, M = 40.17, SE = 2.35, 95% CI [35.43, 44.92] (see Figure 4A). Pairwise comparisons showed that the control and E2R formats did not differ significantly, estimate = −1.779, SE = 1.589, Bonferroni-adjusted 95% CI [−5.631, 2.072], d = −0.18, 95% CI [−0.56, 0.21], *p* = 0.794. The control and hard-to-read formats differed significantly after Bonferroni correction, estimate = −4.297, SE = 1.588, Bonferroni-adjusted 95% CI [−8.146, −0.447], d = −0.43, 95% CI [−0.81, −0.04], *p* = 0.023. The E2R and hard-to-read formats did not differ significantly, estimate = −2.517, SE = 1.582, Bonferroni-adjusted 95% CI [−6.352, 1.318], d = −0.25, 95% CI [−0.63, 0.13], *p* = 0.342. A graphical representation of these Bonferroni-adjusted pairwise comparisons is provided in the Supplementary Materials (Figure S3).

#### 3.3.2. Average Fixation Duration

The best-fitting model retained Text Format as a fixed effect, with random intercepts for Participant and Trial:

Average Fixation Duration ~ Text Format + (1|Participant) + (1|Trial)

Raw average fixation duration was lowest in the E2R format, M = 203 ms, SD = 22.46, followed by the hard-to-read format, M = 216 ms, SD = 21.93, and the control format, M = 219 ms, SD = 25.80. The model showed a significant global effect of Text Format, *p* < 0.001. The likelihood-ratio comparison against the corresponding null model also supported this effect, χ^2^(2) = 46.34, *p* < 0.001. The model showed marginal R^2^ = 0.039 and conditional R^2^ = 0.425.

Estimated marginal means indicated that average fixation duration was lowest in the E2R format, M = 203 ms, SE = 4.61, 95% CI [193.81, 212.64], followed by the hard-to-read format, M = 216 ms, SE = 4.61, 95% CI [206.76, 225.58], and the control format, M = 218 ms, SE = 4.61, 95% CI [209.29, 228.13] (see Figure 4B). Pairwise comparisons showed that the control and E2R formats differed significantly, estimate = 15.490 ms, SE = 2.227, Bonferroni-adjusted 95% CI [10.087, 20.892], d = 0.60, 95% CI [0.39, 0.80], *p* < 0.001. The control and hard-to-read formats did not differ significantly, estimate = 2.542 ms, SE = 2.223, Bonferroni-adjusted 95% CI [−2.854, 7.938], d = 0.10, 95% CI [−0.11, 0.31], *p* = 0.765. The E2R and hard-to-read formats also differed significantly, estimate = −12.947 ms, SE = 2.218, Bonferroni-adjusted 95% CI [−18.327, −7.567], d = −0.50, 95% CI [−0.70, −0.29], *p* < 0.001. A graphical representation of these Bonferroni-adjusted pairwise comparisons is provided in the Supplementary Materials (Figure S4).

#### 3.3.3. Average Saccade Amplitude

The best-fitting model retained the fixed effects of Text Format and Trial Order, with random intercepts for Participant and Trial:

Average Saccade Amplitude ~ Text Format + Trial Order + (1|Participant) + (1|Trial)

Raw average saccade amplitude was greatest in the E2R format, M = 3.48°, SD = 0.56, followed by the hard-to-read format, M = 3.33°, SD = 0.58, and the control format, M = 3.30°, SD = 0.51. The model showed a significant global effect of Text Format, *p* < 0.001, and a significant effect of Trial Order, β = 0.036, SE = 0.011, 95% CI [0.015, 0.056], *p* < 0.001. The model showed marginal R^2^ = 0.019 and conditional R^2^ = 0.724.

Estimated marginal means indicated that average saccade amplitude was greatest in the E2R format, M = 3.49°, SE = 0.11, 95% CI [3.26, 3.72], followed by the hard-to-read format, M = 3.34°, SE = 0.11, 95% CI [3.11, 3.57], and the control format, M = 3.30°, SE = 0.11, 95% CI [3.07, 3.53] (see Figure 4C). Pairwise comparisons showed that the control and E2R formats differed significantly, estimate = −0.189°, SE = 0.038, Bonferroni-adjusted 95% CI [−0.280, −0.098], d = −0.57, 95% CI [−0.84, −0.29], *p* < 0.001. The control and hard-to-read formats did not differ significantly, estimate = −0.040°, SE = 0.038, Bonferroni-adjusted 95% CI [−0.131, 0.051], d = −0.12, 95% CI [−0.39, 0.15], *p* = 0.872. The E2R and hard-to-read formats also differed significantly, estimate = 0.150°, SE = 0.037, Bonferroni-adjusted 95% CI [0.059, 0.240], d = 0.45, 95% CI [0.18, 0.72], *p* < 0.001. A graphical representation of these Bonferroni-adjusted pairwise comparisons is provided in the Supplementary Materials (Figure S5).

## 4. Discussion

The present study examined whether the E2R format, based on visual and typographic adaptations, could influence reading comprehension and visual processing efficiency in young university students without cognitive disabilities. This question is particularly relevant given the ongoing debate about the effectiveness of E2R adaptations, as the empirical evidence supporting E2R recommendations remains limited (González-Sordé et al., 2026), especially for visual and typographic features, as highlighted in a recent systematic review on the topic (Iacono, 2026). In this context, two hypotheses were tested. First, we expected the E2R format to be associated with higher reading comprehension and shorter reading times than the hard-to-read format, or at least with performance comparable to the control format. Second, we expected the E2R format to be associated with shorter fixation durations, fewer fixations, and larger saccade amplitudes than the hard-to-read format, or at least with eye-movement patterns comparable to the control format.

H1 was partially supported. Reading comprehension was significantly affected by Text Format, with estimated accuracy highest in the E2R format and significantly higher than in the hard-to-read format. However, the difference between E2R and control formats showed a trend towards significance. This result is consistent with previous research. For example, higher conformance with accessibility guidelines has been shown to improve performance and user ratings in people without disabilities, as well as in users with visual impairments (Schmutz et al., 2016, 2017). However, reading time showed that E2R format did not differ significantly from the other formats. This more cautious result is in line with Schmutz et al. (2019), who found that E2R language improved some performance outcomes, such as content recognition, but also increased reading time. Thus, accessibility adaptations may improve some outcomes while having weaker or even unfavorable effects on others.

H2 was partially supported. The strongest evidence came from average fixation duration and average saccade amplitude: the E2R format led to shorter fixations and larger saccades than both the control and hard-to-read formats. Thus, it could be argued that this pattern is compatible with reduced processing demands in some eye-movement measures due to reduced visual crowding. In line with previous research on typographic spacing and word recognition (e.g., Perea & Gomez, 2012a, 2012b; Perea et al., 2016), the greater spacing and clearer layout of the E2R format may have facilitated smoother eye-movement behavior. These findings are also consistent with eye-tracking studies showing that E2R adaptations can influence reading behavior. For example, visual support has been shown to facilitate sentence processing in adults with and without dyslexia and in adults with different educational levels (Rivero-Contreras et al., 2021, 2023). However, previous evidence is not uniformly positive: some studies have found that visual support or other E2R recommendations can affect fixation patterns without clearly improving comprehension or perceived difficulty (González-Sordé et al., 2025, 2026). Therefore, the present findings should not be interpreted as evidence that all E2R adaptations are inherently beneficial. Rather, they suggest that the specific combined visual and typographic configuration tested here was associated with a pattern compatible with reduced processing demands in some eye-movement measures, especially when compared with a visually demanding format. However, this interpretation should be considered alongside the more mixed findings observed for fixation count and reading time. 

These findings have both theoretical and practical implications. Theoretically, they contribute to the empirical evaluation of E2R recommendations, a field in which evidence remains limited, heterogeneous, and difficult to generalize (González-Sordé, 2025; González-Sordé & Matamala, 2024; Iacono, 2026; Rivero-Contreras & Saldaña, 2020). Practically, the results suggest that the E2R format may support reading efficiency not only in populations with cognitive or linguistic needs, but also in young adults without cognitive disabilities. This aligns with a universal accessibility perspective, which frames accessible design as the reduction in barriers and the provision of equivalent access for users with diverse abilities, needs, preferences, and contexts of use (Kurt, 2019). Thus, the E2R format may not be understood only as a specialized adaptation, but as part of a broader approach to making written information more accessible to a wide range of readers. This perspective is consistent with recent psychophysiological evidence showing that web accessibility features can support cognitive engagement and perceived readability in users without disabilities (Ekin et al., 2025).

Nevertheless, several limitations should be acknowledged. First, the sample was relatively homogeneous, consisting of university students without cognitive disabilities. Therefore, the generalizability of the findings remains limited. Future studies should include larger and more diverse samples, including older adults, people with dyslexia, individuals with intellectual disabilities, second-language readers, and users with lower literacy levels. In addition, a larger sample size is required. Although the power analysis indicated adequate sensitivity for detecting the main effects observed in comprehension and several eye-movement measures, statistical power was more limited for reading time. Second, the E2R format combined several visual and typographic adaptations at once. This increases ecological validity, because real E2R materials usually combine multiple adaptations, but it prevents us from identifying which specific element drove the effects. Therefore, the observed effects should be interpreted as reflecting the combined E2R format rather than the effect of any single visual or typographic adaptation. Third, the hard-to-read condition may have represented an artificially difficult text format. As a result, differences between the hard-to-read format and the other conditions should be interpreted cautiously, because they may partly reflect the contrast with a challenging presentation rather than the advantage of E2R adaptations over typical real-world materials. Nevertheless, this text format was useful for testing whether visual and typographic presentation can affect reading outcomes under suboptimal conditions. Fourth, the materials were short fictional texts presented in a controlled laboratory setting, which may not fully represent natural reading in educational, administrative, or legal contexts. Finally, the study focused on comprehension and eye movements, but did not include subjective ratings (e.g., of esthetics or perceived effort). Future studies should also include subjective measures such as perceived esthetics, perceived difficulty, or perceived effort, because objective performance benefits may coexist with less favorable user reactions (Schmutz et al., 2019).

## 5. Conclusions

This study examined the impact of an E2R format, based on combined visual and typographic adaptations, on reading comprehension and eye-movement measures while reading in young adults without cognitive disabilities. Within this experimental context, reading comprehension was significantly affected by Text Format, with estimated accuracy highest in the E2R condition and significantly higher than in the hard-to-read condition. Eye-tracking data further showed that the E2R format was associated with shorter fixation durations and larger saccade amplitudes than both the control and hard-to-read formats, suggesting a pattern compatible with a reduced cognitive demand in some eye-movement measures. However, effects on fixation count and reading time were less robust, indicating that the benefits of the E2R format were not uniform across all indicators of reading behavior. Taken together, these findings suggest that, under controlled reading conditions and in a sample of young adults without cognitive disabilities, visual and typographic E2R adaptations may support some aspects of written-information processing. Further research is needed before these findings can be generalized to other populations, longer or more naturalistic texts, and applied educational, health, or legal contexts. Future studies should also examine whether the observed effects of E2R adaptations are maintained across the lifespan, from younger to older readers.

## Data Availability

The materials, datasets and analysis scripts required to reproduce the study are available in the OSF project at https://osf.io/25ar8 (accessed on 12 June 2026).

## Supplementary Materials

The following supporting information can be downloaded at https://www.mdpi.com/article/10.3390/bs16061041/s1, Figure S1: Bonferroni-adjusted pairwise comparisons for reading comprehension; Figure S2: Bonferroni-adjusted pairwise comparisons for reading time; Figure S3: Bonferroni-adjusted pairwise comparisons for fixation count; Figure S4: Bonferroni-adjusted pairwise comparisons for average fixation duration; Figure S5: Bonferroni-adjusted pairwise comparisons for average saccade amplitude.

## Abbreviation

The following abbreviation is used in this manuscript:

E2R	Easy-to-Read
