# Supplementary material for: An Eye-Tracking Study on Text Accessibility and Comprehension in University Students
Source: Behav Sci (Basel). 2026 Jun 22;16(6):1041. doi: 10.3390/bs16061041 (PMC13296324; doi:10.3390/bs16061041)
Supplement: Supplementary file 1 [file behavsci-16-01041-s001.zip › behavsci-4347765-supplementary.pdf]

Supplementary Materials

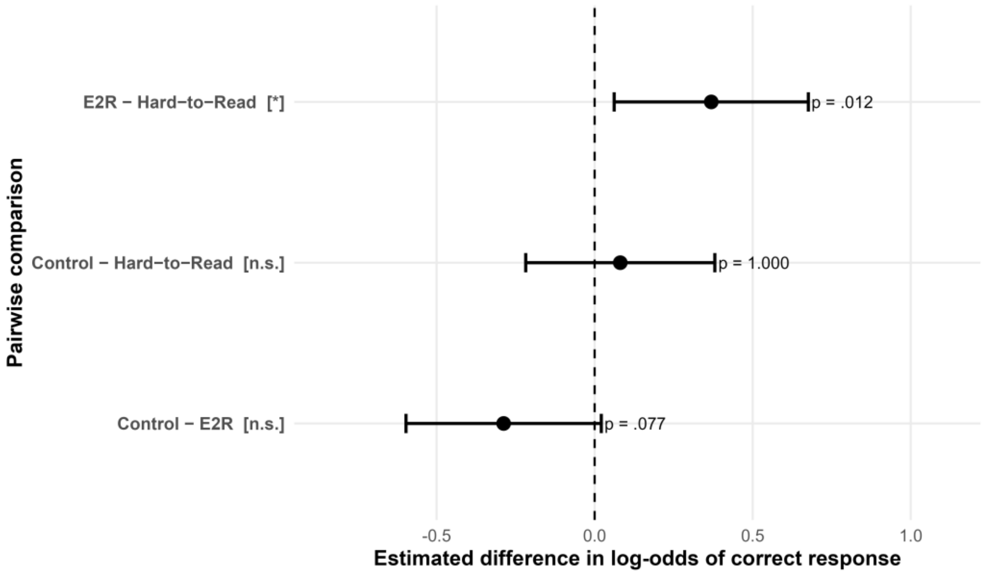

Figure S1. Bonferroni-adjusted pairwise comparisons for reading comprehension. Points represent estimated pairwise differences in reading comprehension, and horizontal error bars represent Bonferroni-adjusted 95% confidence intervals. The vertical dashed line indicates no difference between conditions. Asterisks in square brackets next to each comparison indicate the Bonferroni-adjusted significance level: * p < .05, ** p < .01, *** p < .001; n.s. = non-significant.

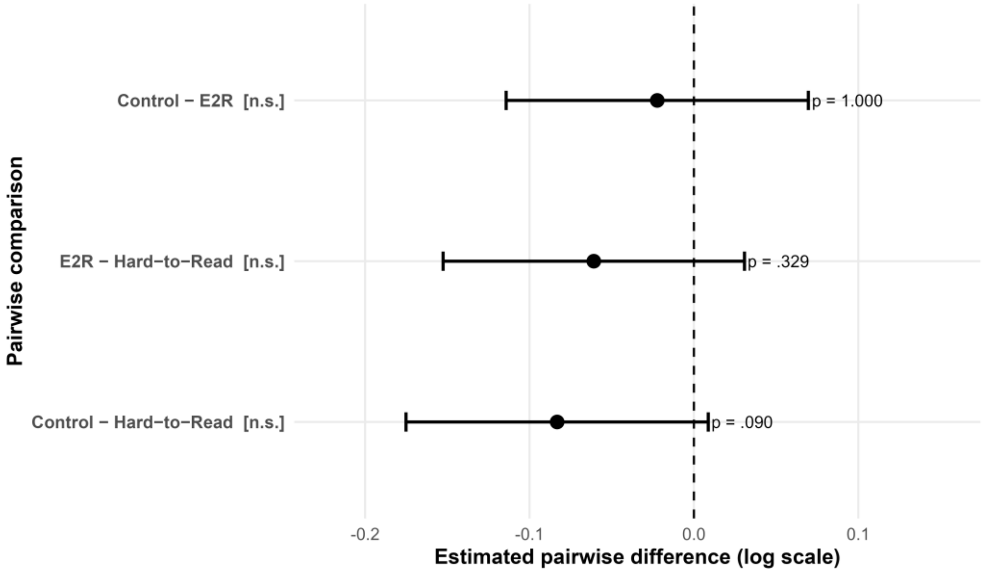

Figure S2. Bonferroni-adjusted pairwise comparisons for reading time. Points represent estimated pairwise differences in reading time on the log-transformed scale, and horizontal error bars represent Bonferroni-adjusted 95% confidence intervals. The vertical dashed line indicates no difference between conditions. Asterisks in square brackets next to each comparison indicate the Bonferroni-adjusted significance level: * p < .05, ** p < .01, *** p < .001; n.s. = non-significant.

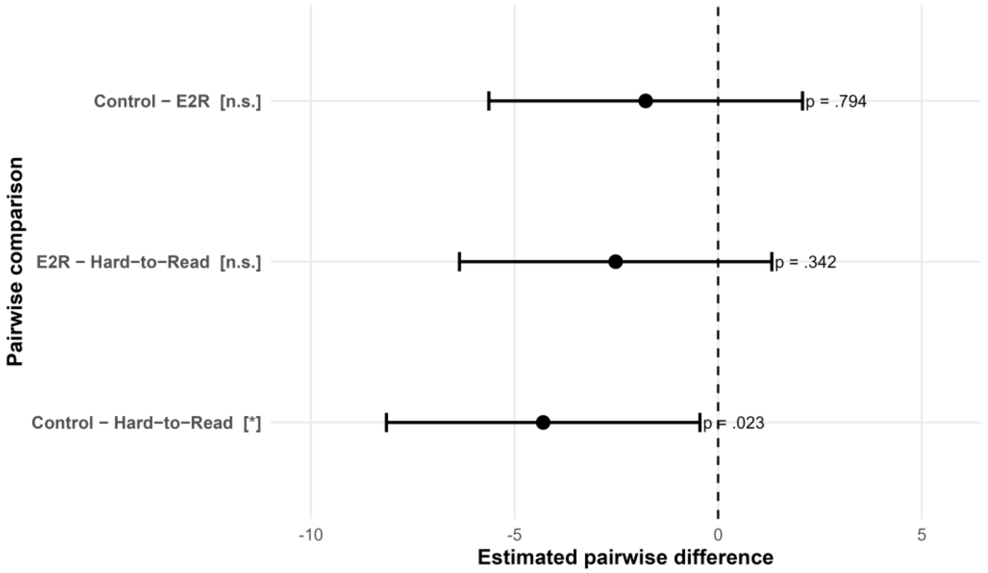

Figure S3. Bonferroni-adjusted pairwise comparisons for fixation count. Points represent estimated pairwise differences in fixation count, and horizontal error bars represent Bonferroni-adjusted 95% confidence intervals. The vertical dashed line indicates no difference between conditions. Asterisks in square brackets next to each comparison indicate the Bonferroni-adjusted significance level: * p < .05, ** p < .01, *** p < .001; n.s. = non-significant.

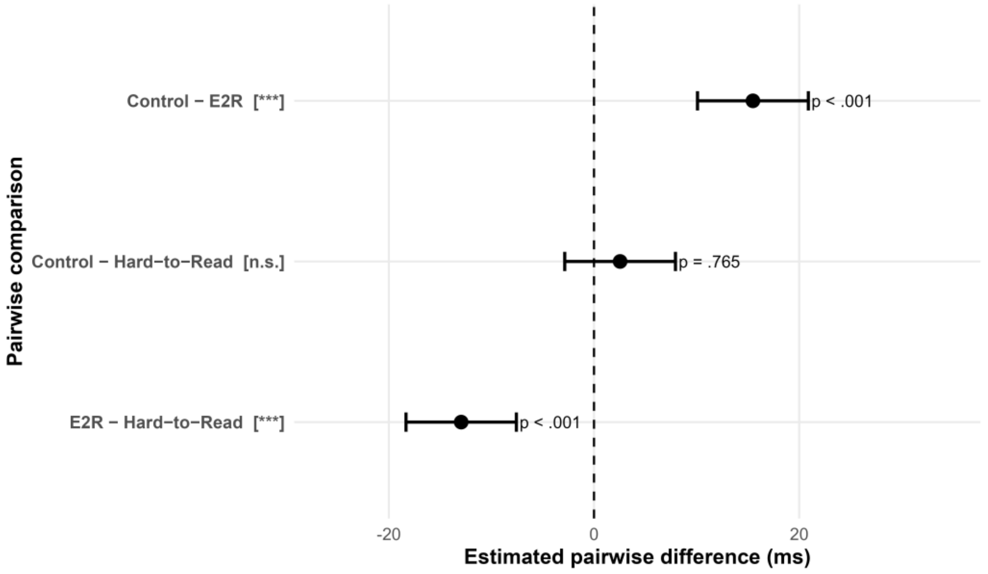

Figure S4. Bonferroni-adjusted pairwise comparisons for average fixation duration. Points represent estimated pairwise differences in average fixation duration, and horizontal error bars represent Bonferroni-adjusted 95% confidence intervals. The vertical dashed line indicates no difference between conditions. Asterisks in square brackets next to each comparison indicate the Bonferroni-adjusted significance level: * p < .05, ** p < .01, *** p < .001; n.s. = non-significant.

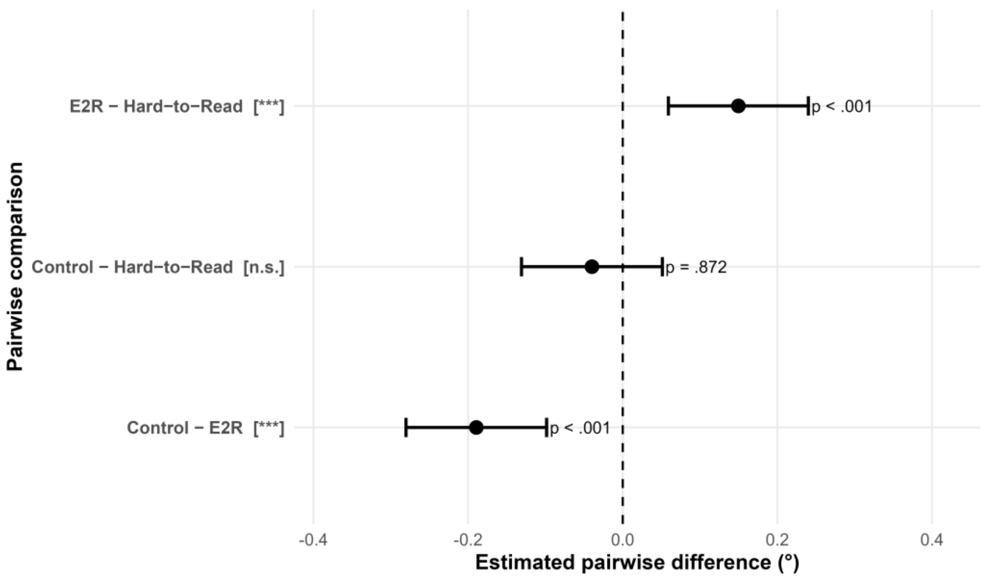

Figure S5. Bonferroni-adjusted pairwise comparisons for average saccade amplitude. Points represent estimated pairwise differences in average saccade amplitude, and horizontal error bars represent Bonferroni-adjusted 95% confidence intervals. The vertical dashed line indicates no difference between conditions. Asterisks in square brackets next to each comparison indicate the Bonferroni-adjusted significance level: * $p < .05$, ** $p < .01$, *** $p < .001$; n.s. = non-significant.
